# Validation status of definitive airway management simulators: a systematic review

**DOI:** 10.1016/j.bja.2026.01.027

**Published:** 2026-03-13

**Authors:** Roisin McCarthy, Liam Maher, Angela O’Dea

**Affiliations:** 1 University of Galway, Galway, Ireland; 2 St. Vincent’s University Hospital, Dublin, Ireland

**Keywords:** airway management, educational measurement, intubation, intratracheal, manikin, simulation training, tracheal intubation, virtual reality

## Abstract

**Background:**

Definitive airway management simulators are widely used for training and evaluating airway management techniques. However, the extent and quality of their validation remain unclear. In this systematic review, we aimed to identify and evaluate studies assessing the validity of airway simulators used in anaesthesia and airway training.

**Methods:**

A comprehensive and systematic literature search was conducted across PubMed, Embase, and Cochrane Library databases up to September 2025, following PRISMA guidelines. Studies were included if they evaluated at least one domain of simulator validity. Methodological quality was assessed using the Joanna Briggs Institute checklist and each simulator was assigned a Level of Recommendation. The review protocol was registered with PROSPERO (CRD420251108102).

**Results:**

From 939 studies screened, 17 articles met the inclusion criteria, and 29 different simulators were included. Validation domains varied widely, with most studies assessing only one or two domains. Predictive validity, linking simulator performance to clinical outcomes, was rarely evaluated. High-fidelity simulators, such as ORSIM® and SimMan®, demonstrated the most comprehensive validation across multiple domains. Validation quality did not consistently correlate with simulator fidelity, and several low-fidelity models demonstrated short-term skill transfer comparable with more complex systems.

**Conclusions:**

Definitive airway simulators are commonly used for training and device evaluation yet are rarely subjected to robust validation. These findings highlight the urgent need for a unified validation framework and higher-quality research to ensure simulators used in airway management accurately reflect clinical realities. This has critical implications for educators, training bodies, and institutions relying on simulators for competency development and device assessment.

**Systematic review protocol:**

PROSPERO (CRD420251108102).

**Editor’s key points**

• Definitive airway management simulators are widely used, but their validation status has not been systematically evaluated.
• This review identifies definitive airway simulators used in training and examines their validation status. It highlights that most are only partially validated and lack robust predictive validation evidence. Levels of recommendation are assigned to each simulator to guide educators and researchers.
• The findings highlight the need for standardised validation frameworks and longitudinal studies to ensure that simulators can reliably improve clinical airway skills.

Airway management is a core competency in anaesthesia practice.^1^^,^^2^ Establishing a definitive airway is regarded as a critical skill and a basic standard of care.^3^ Techniques encompassed within definitive airway management include tracheal intubation, fibreoptic intubation, and front-of-neck access (FONA) procedures. Errors in airway management are associated with significant morbidity and mortality, highlighting the need for effective and structured training. In response, simulation-based training has become widely accepted as a safe and effective method for acquiring and refining complex airway skills in a risk-free environment.^4^ Both the Royal College of Anaesthetists (RCoA) and the College of Anaesthesiologists of Ireland (CAI) endorse simulation as an integral element of anaesthesia education and have integrated it into formal training programmes to promote clinical competence and patient safety.^5^^,^^6^

Definitive airway management simulators are models used to teach and assess the skills required to secure the airway. They may range from low-fidelity bench models to high-fidelity manikins, cadavers, and virtual reality (VR) platforms. Without appropriate validation, airway simulators risk promoting incorrect knowledge or unsafe behaviours and reinforcing incorrect practical techniques. The growing number of available models further complicates efforts to standardise training quality. Feldman and others highlighted significant design flaws as a major contributor to inconsistent validation standards in laparoscopic simulators,^7^ and similar deficiencies have been reported across other surgical simulators.^8^, ^9^, ^10^ Comprehensive validation is essential to ensure simulators replicate key anatomical features and support realistic procedural training with clinical skill transfer.^7^^,^^11^ Despite their widespread use in training, the extent and quality of validation for definitive airway simulators remain unclear, raising concerns regarding their reliability, educational impact, and suitability for standardised assessment.

Validation studies assess whether an airway simulator adequately replicates relevant anatomical and procedural elements of airway management and whether practice using the simulator is associated with improved clinical practice.^11^ These studies typically examine one or more forms of validity. One example of comprehensive simulator validation is a study evaluating the RealSpine® high-fidelity spinal surgery simulator.^12^ This study exemplifies a comprehensive validation approach and highlights the standard that airway simulator validation should strive to meet. Importantly, no such study has been conducted in the context of airway management simulators.

The aim of this review is to identify definitive airway management simulators described in the literature, evaluate the validity evidence supporting them, and assign levels of evidence (LoEs) and levels of recommendation (LoRs) based on these findings.

## Methods

**Fig 1:**
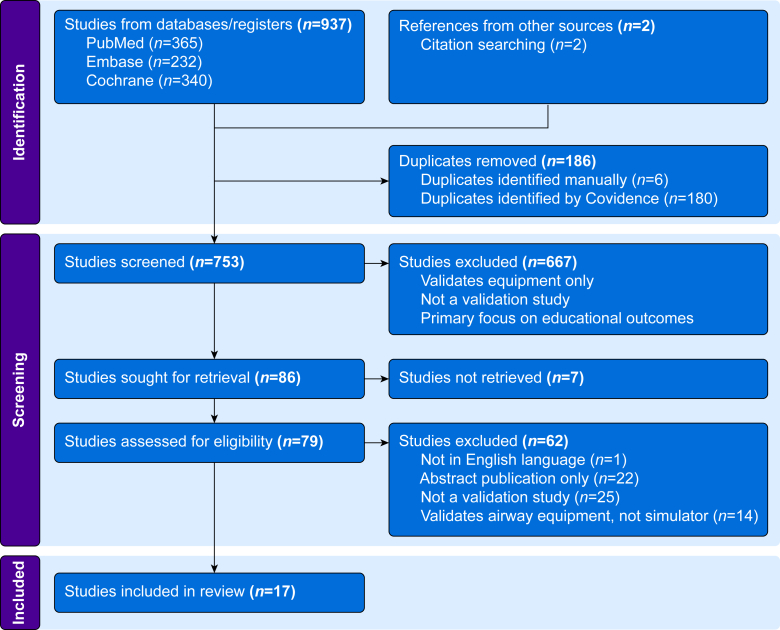
PRISMA flowchart.

This systematic review was conducted in accordance with the Preferred Reporting Items for Systematic Reviews and Meta-Analysis (PRISMA) guidelines (Fig. 1).^13^ The review protocol was registered with PROSPERO (CRD420251108102).

### Information sources and search

PubMed, EMBASE, and the Cochrane Library were searched for articles describing definitive airway management simulators. Each database was searched from its earliest available records to September 2025. The reference lists of included studies were also screened systematically. Search terms included combinations of synonyms for ‘airway’, ‘simulator’, and ‘validation’. The complete search strategies for each database are provided in Supplementary material 1.

### Study eligibility criteria and selection

Articles evaluating or validating an airway simulator used for definitive airway management, including tracheal intubation, fibreoptic intubation, and FONA, were included. Eligible studies reported empirical data on simulator validity including face, content, construct, concurrent, or predictive validity (Table 1). Eligible simulators included adult and paediatric models, comprising dry-lab manikins, task trainers, VR systems, and cadaveric human airway models. Exclusion criteria comprised non-empirical studies, non-English publications, articles describing only the initial design stages of simulators or software, and studies using cadaveric animal models. All records were imported into Covidence, which automatically identified and removed duplicates. The remaining records were screened independently by two reviewers, who identified and removed additional duplicates. Discrepancies at any stage of screening or data extraction were resolved by consensus and if necessary, by consulting a third reviewer.

**Table 1:** Validity Definitions and Assessment based on Van Nortwick and colleagues^14^ and McDougall.^11^ I-CVI, Item Content Validity Index; OSATS, Objective Structured Assessment of Technical Skills.

Validity type	Definition	Validity assessment examples
Face validity	Pertains to the degree to which a simulator appears realistic and credible to non-experts	Subjective assessment • Likert rating scale • Binary response (realistic/not realistic)
Content validity	Determines if the simulator effectively teaches what it is designed to teach as evaluated by subject matter experts	Subjective assessment • Expert panel ratings • Delphi Consensus • I-CVI ≥0.80^15^
Construct validity	This measures the simulator's ability to differentiate between levels of expertise	Statistical difference between groups • Measures: task time, errors, OSATS scores
Concurrent validity	The degree to which the simulator correlates with a gold standard	Anatomical fidelity • Measurements: dimensions/angles Positive correlation with a gold standard
Predictive validity	The extent to which performance on the simulator predicts future performance in real clinical settings	Longitudinal correlation with real clinical performance : • Success rates, time to intubation, error reduction, complication rates

### Data extraction

After initial screening, eligible studies underwent full-text review. Data were extracted systematically using a structured Microsoft Excel® spreadsheet (Microsoft Corporation, Redmond, WA, USA), capturing variables including authorship, publication year, study design, study context, simulator type and fidelity, validation type, assessment methods, and outcomes measured (Table 2). Commercial availability of simulators was also recorded. Fidelity classification was standardised to ensure consistency across studies. We applied a framework based on the physical, functional, and procedural fidelity of each simulator with respect to airway management.^16^ Low-fidelity simulators were defined as static models that lack realistic anatomy, tissue responsiveness, or physiological feedback to airway management, such as a task trainer. High-fidelity simulators were defined as platforms that incorporated advanced anatomical realism, tissue responsiveness, or physiological feedback to airway management. These included physiological responsive manikins, cadaveric models, and VR systems. The extraction form was piloted on three studies to ensure usability. Missing data were classified as ‘not specified’.

**Table 2:** Simulator types, simulator fidelity, describing studies, study characteristics, simulator validity, study characteristics, study LoE, and simulator LoR. LoE, level of evidence; LoR, level of recommendation; N, no; Y, yes.

Simulator model (manufacturer)	Simulator type	Simulator fidelity	First author (year)	Participants		Validity type	Validation outcome	LoE	LoR
				*n*	Demographics				
Premature Anne® Neonatal Manikin (Laerdal Medical®, Stavanger, Norway)	Manikin	High	Rumpel (2023)^17^	102	Novices (paediatric trainees	Predictive	N	2c	3
						Construct	Y		

TruCorp® AirSim Trainer (TruCorp Belfast, UK)	Modified manikin	Low (with modifications)	Brettig (2017)^18^	130	Experts, novices (consultant anaesthetists, anaesthesia trainees, students)	Construct Face	Y N	2b	3
• Modified to simulate three difficult airways									

TruCorp® AirSim Trainer (TruCorp®, Belfast, Ireland)	Manikin	Low	Schalk (2015)^19^	47	Human upper airway CT scans	Concurrent	N	2b	2
			Blackburn (2021)^20^	33	Human upper airway CT scans	Concurrent	N	2b	

SimMan® Simulator (Laerdal Medical®, Stavanger, Norway)	Manikin	High	Hesselfeldt (2005)^21^	60	Experts, novices (consultant anaesthetists, nurse anaesthetists, anaesthesia trainees)	Face	Partially Y	2c	2
			Schebesta (2012)^22^	20	Human upper airway CT scans	Concurrent	N	2b	
			Schebesta (2015)^23^	40	Novices (anaesthesia trainees)	Concurrent Face	Y Y	2c	
			Schalk (2015)^19^	47	Human upper airway CT scans	Concurrent (anatomical)	Y	2b	

Fix for Life (F4L) cadaver	Cadaver	High	van Emden (2018)^24^	30	Experts, novices (consultant anaesthetists, anaesthesia trainees)	Face	Y	2c	3
						Content	Y		

Formalin-fixed cadavers	Cadaver	High	van Emden (2018)^24^	30	Experts, novices (consultant anaesthetists, anaesthesia trainees)	Face	N	2c	3
						Content	N		

SimMan 3G® (Laerdal Medical®, Stavanger, Norway)	Manikin	High	van Emden (2018)^24^	30	Experts, novices (consultant anaesthetists, anaesthesia trainees)	Face Content	Y Y	2c	2
			Schebesta (2012)^22^	20	Human upper airway CT scans	Concurrent (anatomical)	N	2b	

HPS Human Patient Simulator® (METI®, Sarasota, FL, USA)	Manikin	High	Schebesta (2012)^22^	20	Human upper airway CT scans	Concurrent (anatomical)	Partially Y	2b	2
			Schalk (2015)^19^	47	Human upper airway CT scans	Concurrent (anatomical)	Y	2b	

HAL S3000® Mobile Team Trainer (Gaumard®, Miami, FL, USA)	Manikin	High	Schebesta (2012)^22^	20	Human upper airway CT scans	Concurrent (anatomical)	N	2b	2
			Schebesta (2015)^23^	40	Novices (anaesthesia trainees)	Concurrent (procedural)	Y	2b	
Laerdal Airway Management Trainer® (Laerdal Medical®, Stavanger, Norway)	Manikin	Low	Schebesta (2012)^22^	20	Human upper airway CT scans	Concurrent (anatomical)	N	2b	2
			Schalk (2015)^19^	47	Human upper airway CT scans	Concurrent (anatomical)	Y	2b	
			Blackburn (2021)^20^	33	Human upper airway CT scans	Concurrent (anatomical)	N	2b	

AMBU M MegaCode Trainer W® (AMBU®, Ballerup, Denmark)	Manikin	Low	Schebesta (2012)^22^	20	Human upper airway CT scans	Concurrent (anatomical)	N	2b	3

Laerdal Resusci Anne Simulator® (Laerdal Medical®, Stavanger, Norway)	Manikin	Low	Schalk (2015)^19^	47	Human upper airway CT scans	Concurrent (anatomical)	Y	2b	3

VBM Atemwegssimulator Bill® (VBM®, Sulz-Neckar, Germany)	Manikin	Low	Schalk (2015)^19^	47	Human upper airway CT scans	Concurrent (anatomical)	N	2b	3

Gaumard Scientific NOELLE Birthing Simulator® (Gaumard®, Miami, FL, USA)	Manikin	Low	Schalk (2015)^19^	47	Human upper airway CT scans	Concurrent (anatomical)	N	2b	3

AMBU® Intubationstrainer Erwachsene (AMBU®, Bad Nauheim, Germany)	Manikin	Low	Schalk (2015)^19^	47	Human upper airway CT scans	Concurrent (anatomical)	N	2b	3

AMBU® MegaCode W (Bad Nauheim, Germany)	Manikin	Low	Schalk (2015)^19^	47	Human upper airway CT scans	Concurrent (anatomical)	N	2b	3

VBM Atemwegssimulator Bob® (VBM®, Sulz-Neckar, Germany)	Manikin	Low	Schalk (2015)^19^	47	Human upper airway CT scans	Concurrent (anatomical)	N	2b	3

AMBU Airway Man I® (AMBU®, Bad Nauheim®, Germany)	Manikin	Low	Schalk (2015)^19^	47	Human upper airway CT scans	Concurrent (anatomical)	N	2b	3

Laerdal Resusci Anne Advanced Skilltrainer® (Laerdal Medical®, Stavanger, Norway)	Manikin	Low	Schalk (2015)^19^	47	Human upper airway CT scans	Concurrent (anatomical)	Y	2b	3

Laerdal MegaCode Kelly ALS® (Laerdal Medical®, Stavanger, Norway)	Manikin	Low	Schalk (2015)^19^	47	Human upper airway CT scans	Concurrent (anatomical)	N	2b	3

3D printed airway manikins with a virtual reality simulation system	Hybrid virtual reality+manikin	High (hybrid simulator)	Jain (2025)^25^	7	Experts (four consultant intensivists, three emergency medicine consultants)	Face	Y	3	3

SynDaver® Standard Adult Airway Trainer (SynDaver Labs®, Tampa, FL, USA)	Manikin	Low	Blackburn (2021)^20^	33	Human upper airway CT scans	Concurrent (anatomical)	N	2b	3

‘High-fidelity computerised infant manikin’ (manufacturer not specified)	Manikin	High	Finan (2012)^26^	13	Novices (paediatric trainees)	Predictive	N	2c	3

Fibreoptic intubation training simulators									

Partial Task Trainer Box with polymer baffles	Bench	Low	Williams (2010)^27^	75	Experts, novices (consultant anaesthetists, students)	Construct Face	Y Y	3	4

‘Choose-the-hole’ model (University of Toronto, Toronto, ON, Canada)	Bench	Low	Naik (2001)^28^	24	Novices (anaesthesia trainees, internal medicine residents)	Predictive	Y	2b	3

EndoVR® GI and Bronchoscopy Simulator (CAE Healthcare®, Sarasota, FL, USA)	Virtual reality	High	Samuelson (2016)^29^	33	Novices (anaesthesia trainees)	Predictive (warm-up)	Y	2b	3

ORSIM® bronchoscopy simulator (Airway Simulation Limited®, Auckland, New Zealand)	Virtual reality	High	Wong (2019)^30^	34	Novices (anaesthesia trainees, anaesthesia assistants, students)	Predictive	Y	1b	2
			Baker (2016)^31^	28	Experts, novices (consultant anaesthetists, anaesthesia trainees, anaesthesia technicians)	Construct	Y	2b	

AccuTouch® Flexible Bronchoscopy Simulator (Immersion Medical Systems®, Gaithersburg, MD, USA)	Virtual Reality	High	Rowe (2002)^32^	20	Novices (paediatric trainees)	Predictive	Y	2b	3

Front-of-neck access training simulators									

3D-printed Paediatric Airway model	Modified manikin	Low (with modifications)	Kovatch (2020)^33^	6	Six experts (three anaesthetists, three otolaryngologists)	Content	Y	4	4
• Incorporated into a basic commercial paediatric head and neck manikin									

### Risk of bias and quality appraisal

Methodological quality and risk of bias were assessed independently by two reviewers using the appropriate Joanna Briggs Institute (JBI) critical appraisal tool for each study design (Supplementary material 2).^34^ Any discrepancies were resolved by consensus or, if necessary, through consultation with a third reviewer.

In addition, a modified educational framework based on the Oxford Centre for Evidence-Based Medicine (OCEBM) and adapted by the European Association of Endoscopic Surgery was applied to determine each study's LoE.^35^^,^^36^ LoE ranges from Level 1, representing the highest level of evidence, to Level 4, the lowest. The LoE informed the corresponding LoR assigned to each simulator (Table 2, Supplementary material 3).

### Data analysis

Simulators were categorised into three groups based on the airway management skill they were designed to assess: (i) tracheal intubation, (ii) fibreoptic intubation, and (iii) FONA simulators. Validation types were defined based on frameworks proposed by Van Nortwick and colleagues^14^ and McDougall^11^ (Table 1). An LoR score was assigned to each simulator with Level 1 LoR representing the strongest recommendation for a simulator, and Level 4, the weakest (Table 2).

Given the substantial heterogeneity in both study design and measured outcomes, a narrative synthesis approach was used to examine emerging patterns, trends, and evidence gaps across the dataset. This method allowed contextualisation of findings without statistical pooling. Results were synthesised using a categorisation framework by simulator type, with subcategories for model design and validation approach. Key characteristics and LoR ratings are presented in tabular format using Microsoft Excel® (Microsoft Corporation; Table 2).

## Results

Of the 939 articles retrieved from the search, 17 articles met the inclusion criteria which directly evaluated or validated a simulator (Fig. 1). Many studies were excluded during abstract screening as they focused on airway devices, general simulation training, or educational outcomes rather than evaluating or validating the simulators themselves. The remaining simulators were categorised according to the airway management skill they were designed to assess tracheal intubation (*n*=23), fibreoptic intubation (*n*=5), and FONA (*n*=1) (Table 2). Table 3 summarises the distribution of simulator model types, fidelity levels, validity domains and LoR for the included simulators.

**Table 3:** Frequency table summarising simulator types, simulator fidelity, validity domains, and levels of recommendation across included studies. ∗Percentages calculated from 29 simulator evaluations across 17 included studies.

Category	Subcategory	*n* (%∗)	Examples
Simulator type	Tracheal intubation	23 (79)	SimMan®, HPS®, TruCorp AirSim® Fix for Life cadaver
	Fibreoptic intubation	5 (17)	ORSIM®, EndoVR®, ‘choose-the-hole’ bench model
	Front-of-neck access (FONA) simulators	1 (3)	3D-printed paediatric cricothyrotomy model
Fidelity level	Low fidelity	17 (59)	Bench trainers, basic airway manikins
	High fidelity	12 (41)	SimMan®, HPS®, ORSIM®, AccuTouch®, EndoVR®
Validity type assessed	Face validity	7 (24)	Subjective realism ratings
	Content validity	4 (14)	Expert evaluation of educational relevance
	Construct validity	4 (14)	Expert–novice discrimination studies
	Concurrent validity	17 (59)	Correlation to gold standard (such as human anatomy)
	Predictive validity	6 (21)	Correlation with clinical or performance outcomes
Level of recommendation (LoR)	2	7 (24)	SimMan®, HPS®, ORSIM®
	3–4	22 (76)	AMBU® M MegaCode, AccuTouch®

### Tracheal intubation training simulators

Ten studies assessed the validity of 23 airway management simulators for tracheal intubation training. Of these, nine were high-fidelity and 14 were low fidelity. The majority (20 studies) were manikin based, with two cadaveric models and one VR simulator combined with a manikin; the latter was the only VR model to meet inclusion criteria (Table 2). Eighteen simulators were commercially available, two were newly developed, and two were cadavers; one study did not identify the simulator at all, despite reporting on predictive validity.^26^ No study completely validated a simulator across all dimensions of validity and most studies assessed only one type of validity, most commonly concurrent validity, typically by comparing simulators with anatomical or clinical gold standards (Table 2). Two studies assessed predictive validity, and both reported no improvement in real-world neonatal intubation outcomes after simulator training.^17^^,^^26^

Only three simulators were assessed for more than one type of validity. SimMan® (Laerdal Medical®, Stavanger, Norway) was the most extensively validated simulator, appearing in four independent studies assessing face, content, and concurrent validity, including one RCT.^19^^,^^21^^,^^22^^,^^23^ The SimMan 3G**®** (Laerdal Medical®) was assessed for face, content, and concurrent validity across two independent studies (both LoE 2b), supporting an overall LoR of 2.^22^^,^^24^ The same principle was applied to each simulator and is summarised in Table 2.

Cadaveric studies revealed variation in performance between formalin-fixed and Fix for Life embalmed cadaver models, with the latter rated higher for realism and teaching value.^24^ One study investigated a modified TruCorp AirSim® (TruCorp®, Belfast, Ireland) simulator that incorporated adjustable airway difficulty, demonstrating both construct and face validity.^18^ Finally, one hybrid model integrating VR with a manikin demonstrated high face validity but lacked comprehensive evaluation.^25^

### Fibreoptic intubation training simulators

Six studies evaluated the validity of five airway management simulators used for fibreoptic intubation training. Two were low-fidelity bench models, which are not commercially available, whereas the remaining three were high-fidelity VR simulators, all of which are commercially available (Table 2). Four of the five simulators were assessed for predictive validity, indicating comparatively stronger evidence for real-world performance than that found for the tracheal intubation simulators (Table 2).

Among high-fidelity VR simulators, the ORSIM® (Airway Simulation Limited®, Auckland, New Zealand) underwent the most comprehensive validation, being evaluated across two studies.^30^^,^^31^ One study demonstrated construct validity, showing that experts consistently outperformed novices and trainees in both speed and blinded performance scores, with high inter-rater reliability (>0.8).^31^ Another study provided evidence of predictive validity, showing that simulator performance correlated with clinical performance in novices.^30^

The AccuTouch® simulator (Immersion Medical Systems®, Gaithersburg, MD, USA) was also evaluated for predictive validity.^32^ After simulator training, paediatric residents with no previous experience demonstrated significant improvements in live fibreoptic intubations, including reduced time to intubation (from 5.15 min to 0.88 min) and fewer mucosal collisions. The EndoVR® simulator (CAE Healthcare®, Sarasota, FL, USA) was assessed as a virtual warm-up tool immediately before live intubation, with participants in the warm-up group outperforming controls.^29^

Two studies investigated low-fidelity, non-immersive models for fibreoptic intubation training, both assessing skill acquisition and transfer into clinical performance.^27^^,^^28^ One used a ‘choose-the-hole’ bench model to train fibreoptic manipulation skills and demonstrated predictive validity. In this RCT, participants who trained on the model significantly outperformed those receiving didactic teaching alone in a real clinical environment.^28^ Another study evaluated a more anatomically realistic manikin but reported only face and content validity based on user feedback.^27^

Despite these examples, overall validation of fibreoptic intubation simulators remained limited.

### Front-of-neck access training simulators

Only one validation study for FONA met the inclusion criteria.^33^ This study evaluated a 3D-printed paediatric airway for cricothyroidotomy integrated into a basic commercial paediatric head and neck manikin. However, the specific manikin that was modified was not identified. The modified simulator demonstrated high visual and anatomical fidelity and received positive expert ratings across domains of realism, value, and usability. Validation was limited to content validity based on expert opinion (*n*=6), with no assessment of construct or predictive validity, no control groups, and no evaluation of clinical performance outcomes.

## Discussion

This systematic review identified 17 articles evaluating a range of definitive airway management simulators, most of which were low-fidelity, manikin-based models. The majority assessed only one dimension of validity, typically concurrent anatomical validity, and only a small subset examined predictive validity. LoE varied, with most studies rated at Level 2b or 2c and only a few simulators achieving LoR scores of lower than 3. Most of the studies included in this review did not state the method of validation assessed.

### Tracheal intubation training simulators

Validation of tracheal intubation simulators was inconsistent across included studies and was largely limited to early stages of validity, primarily face or concurrent validation assessments. No simulator underwent comprehensive validation. Predictive validity, the most clinically relevant measure, was rarely assessed.

The repeated finding of anatomical discrepancies between manikins and human imaging data^19^^,^^20^^,^^22^ challenge their assumed educational realism and highlight the need for independent verification of manufacturers’ claims of anatomical accuracy.^37^^,^^38^ In contrast, a later study reported acceptable functional performance on the same simulators with procedural outcomes comparable with real patients.^23^ This highlights an important conceptual issue: simulators that lack anatomical realism may still support effective skill acquisition. This distinction highlights the importance of context when interpreting different aspects of validity and that educational fidelity may sometimes be more relevant than perfect anatomical replication.

Cadavers are often regarded as the validation gold standard in airway simulators owing to their anatomical authenticity.^39^ However, recent evidence suggests notable variation in realism depending on tissue preservation methods, suggesting that not all cadavers provide equal educational value.^24^ The more pliable Fix for Life model outperformed the formalin-fixed cadavers, indicating that even cadaveric validation requires careful methodological consideration.^24^

There was an absence of demonstrable predictive validity in two intubation simulator studies, despite improved intubation performance on the simulators themselves.^17^^,^^26^ Therefore, improved simulator performance does not necessarily equate to improved clinical outcomes. Both studies assessed neonatal intubation, which is an inherently challenging skill and may partly explain the limited skill transferability.^40^ Emerging hybrid and VR systems offer dynamic feedback and modifiable difficulty, creating a more realistic training experience.^25^ However, these technologies remain under-validated with few comparative or longitudinal studies available.

Overall, although SimMan® and the HPS Human Patient Simulator® achieved moderate validation (LoE 2b; LoR 2), methodological heterogeneity, small sample sizes, and the limited scope of existing studies restrict the strength of current conclusions.

### Fibreoptic intubation training simulators

Evidence supporting fibreoptic intubation simulators is methodologically stronger than for tracheal intubation simulators, with more studies assessing predictive validity and using objective performance metrics. The predominance of VR-based simulators for fibreoptic intubation training likely reflects the visual and navigational nature of the skill.^41^ However, most validation remains incomplete, limited to short-term performance gains rather than sustained clinical competence.

Among high-fidelity VR models, the ORSIM® demonstrates the most comprehensive validation.^30^^,^^31^ Studies demonstrated strong construct validity and predictive validity.^30^^,^^31^ Inclusion of both normal and difficult airway scenarios strengthens its educational relevance, although the brief intervals between simulation and clinical assessment, typically <1 week, limit these conclusions to short-term skill transfer. Similarly, the AccuTouch® and EndoVR® simulators demonstrated improved clinical performance immediately after simulator training.^29^^,^^32^ These improvements may represent procedural priming rather than sustained skill acquisition. Consequently, predictive validity derived from immediate post-training clinical performance should be interpreted with caution.

Low-fidelity models can also support clinical skill transfer. The simple ‘choose-the-hole’ bench model demonstrated predictive validity in a RCT,^28^ although clinical testing once again occurred within 1 week of simulator training. Conversely, a low-fidelity simulator made from polymer baffles achieved only face and content validity based on user feedback, with no objective metrics or control group for comparison.^27^

Overall, although high-fidelity VR simulators such as ORSIM® demonstrate the strongest evidence for predictive validity, their educational superiority over simpler models has not yet been demonstrated.

### Front-of-neck access training simulators

The single eligible study highlights both innovation and limitation in the current FONA simulation literature.^33^ Its reliance on expert opinion and subjective content, without objective performance metrics, comparative groups, or assessment of skill transfer, substantially limits the robustness and generalisability of its findings.

Unlike intubation or fibreoptic techniques, real-world predictive validity cannot be ethically or practically evaluated for FONA procedures. Consequently, FONA simulator validation may need to rely on demonstrated improvements and skill retention within simulated or cadaveric environment, rather than on clinical outcomes. This highlights the underrepresentation of FONA simulators and the need to address this gap to support competence in rare, life-saving procedures.

### Current literature

Unlike simulation practices in high-stakes industries such as aviation and nuclear energy, where strict and regularly updated standards govern simulator design and performance, medical simulation remains largely unregulated. Concerns about the absence of formal validation frameworks were raised more than a decade ago, warning that unstandardised simulation undermines educational reliability and comparability.^42^ Despite recommendations for structured frameworks based on interactivity, physiological modelling, and reproducibility, unified validation criteria are still lacking. A recurring issue in the airway literature is the assumption that results from unvalidated simulators can be generalised to clinical performance. For example, studies comparing airway devices using static manikin models have drawn clinical conclusions without verifying anatomical accuracy or transferability of these manikins to clinical practice.^43^, ^44^, ^45^, ^46^

### Strengths and limitations

This systematic review used a comprehensive search strategy across multiple databases and backward citation tracking to maximise identification of relevant studies. Independent screening and data extraction, combined with structured critical appraisal tools, enhanced methodological transparency and consistency.

Despite the strengths of this systematic review, several limitations should be acknowledged. Restricting inclusion to English-language, published studies may have introduced language and publication bias. Variability in terminology across disciplines may have resulted in missed studies despite the broad search strategy. The reliance on title and abstract fields may also have limited the retrieval of studies. Evidence for FONA simulators was notably limited, with only one low-quality study meeting inclusion criteria.^33^ Although this reflects a genuine deficiency in the literature, it also raises the possibility that relevant studies were missed despite a comprehensive search strategy. Most simulators lacked high-quality, independently replicated validation, limiting the strength of the LoR applied. Finally, elements of subjective judgement inherent to narrative synthesis may have introduced reviewer bias.

### Future research and implications for practice

The included studies highlight several methodological and educational limitations in current airway simulator validation evidence. Many studies were small, cross-sectional, and underpowered, with inconsistent definitions of user expertise, making comparisons difficult. Some studies lacked essential methodological detail, including simulator identification, which limits interpretation of skill transfer findings.^26^ Most studies relied on subjective assessments of face or construct validity, with few extending to predictive validity or evaluating long-term skill retention. When predictive validity was assessed, follow-up intervals were typically short, restricting conclusions about durable skill transfer.

Future research should prioritise adequately powered, standardised and longitudinal validation studies that use objective performance measures and clearly defined participant groups. Randomised designs comparing simulator training with alternative educational methods and assessment of real-world performance outcomes are required to determine whether simulator-acquired skills translate to clinical proficiency. From a practical perspective, educators and training institutions should exercise caution when interpreting simulator performance as evidence of clinical competence. Validation evidence should guide the selection and endorsement of simulators for teaching and assessment. The development of a unified, transparent validation framework, supported by professional and academic consensus, will be essential to improving simulator design, comparability, and clinical relevance.

## Conclusions

This systematic review demonstrates that definitive airway simulators are widely used but infrequently undergo rigorous, standardised validation. Most studies validate only early validity domains, with limited evidence for predictive or concurrent validity, reducing confidence in their ability to support clinical training.

Moving forward, there is an urgent need for a unified, transparent validation framework that applies consistent terminology and incorporates measurable, standardised criteria. Future research should prioritise predictive and long-term validity to establish whether simulator-based learning translates to sustained clinical competence. Only through rigorous, evidence-based validation can simulators reliably support high-quality training, assessment and safe clinical practice in anaesthesia.

## Authors' contributions

Study design, article screening, data collection, narrative review: RM

Second reviewer: LM

Academic supervisor: AOD

## Declaration of interest

The authors declare that they have no conflicts of interest.
