# Supplementary material for: Validation status of definitive airway management simulators: a systematic review
Source: Br J Anaesth. 2026 Mar 13;136(5):1595–605. doi: 10.1016/j.bja.2026.01.027 (PMC13197900; doi:10.1016/j.bja.2026.01.027)
Supplement: Supplementary file 1 [file mmc1.docx]

**Appendices**

Appendix 1: Search Strategy

**Cochrane Library**

#1 MeSH descriptor: [Virtual Reality] 1 tree(s) exploded

#2 MeSH descriptor: [Models, Anatomic] 1 tree(s) exploded

#3 MeSH descriptor: [Models, Structural] this term only

#4 MeSH descriptor: [Cadaver] this term only

#5 (Simulator:ti,ab OR Mannequin:ti,ab OR Manikin:ti,ab OR Cadaver:ti,ab OR "Virtual Reality":ti,ab OR Haptic:ti,ab OR Task Trainer:ti,ab OR Trainer:ti,ab OR Cricothyroidotomy:ti,ab OR Cricothyrotomy:ti,ab)

#6 ("fiberoptic intubation":ti,ab OR "fiberoptic intubation":ti,ab OR "fibre-optic intubation":ti,ab OR "fiber-optic intubation":ti,ab OR "endotracheal intubation":ti,ab OR "fibreoptic":ti,ab OR "fiberoptic":ti,ab OR intubation:ti,ab)

#7 MeSH descriptor: [Intubation, Intratracheal] this term only

#8 MeSH descriptor: [Intubation, Intratracheal] this term only

#9 MeSH descriptor: [Fiber Optic Technology] this term only

#10 (Validat*:ti,ab OR Validity:ti,ab OR Evaluat*:ti,ab)

#11 MeSH descriptor: [Evaluation Studies as Topic] this term only

#12 MeSH descriptor: [Validation Studies as Topic] this term only

#13 MeSH descriptor: [Reproducibility of Results] 2 tree(s) exploded

#14 (#1 OR #2 OR #3 OR #4 OR #5) AND (#6 OR #7 OR #8 OR #9) AND (#10 OR #11 OR #12 OR #13)

**PubMed**

("Virtual Reality"[MeSH Terms] OR "models, anatomic"[MeSH Terms] OR "Manikins"[MeSH Terms] OR "Cadaver"[MeSH Terms] OR "Simulator"[Title/Abstract] OR "mannequin"[Title/Abstract] OR "manikin"[Title/Abstract] OR "Cadaver"[Title/Abstract] OR "Virtual Reality"[Title/Abstract]) AND ("intubation, intratracheal/methods"[MeSH Terms] OR "intubation, intratracheal/instrumentation"[MeSH Terms] OR "fiber optic technology/education"[MeSH Terms] OR "fiber optic technology/instrumentation"[MeSH Terms] OR "fiberoptic intubation"[Title/Abstract] OR "fiber-optic intubation"[Title/Abstract] OR "fibre-optic intubation"[Title/Abstract] OR "fiber-optic intubation"[Title/Abstract] OR "endotracheal intubation"[Title/Abstract] OR "cricothyroidotomy"[Title/Abstract]) AND ("Reproducibility of Results"[MeSH Terms] OR "Validation Study"[Publication Type] OR "Validation Studies as Topic"[MeSH Terms] OR "validat*"[Title/Abstract] OR "Validity"[Title/Abstract] OR "Evaluation Studies as Topic"[MeSH Terms] OR "Evaluation Study"[Publication Type] OR "Evaluation"[Title/Abstract])

**Embase**

('endotracheal intubation'/exp OR 'endotracheal intubation':ti,ab OR 'fiberoptic intubation'/exp OR 'fiberoptic intubation':ti,ab OR 'fibreoptic intubation'/exp OR 'fibreoptic intubation':ti,ab OR 'endotracheal':ti,ab OR 'intubation':ti,ab OR 'tracheotomy':ti,ab) AND ('simulator'/exp OR 'simulator':ti,ab OR 'virtual reality'/exp OR 'virtual reality':ti,ab OR 'mannequin'/exp OR 'manikin'/exp OR 'manikin':ti,ab OR 'cadaver'/exp OR 'cadaver':ti,ab) AND ('validation study'/exp OR 'evaluation':ti,ab OR 'validity':ti,ab) AND ('article'/it OR 'clinical trial'/it OR 'review'/it)

Appendix 2: JBI Critical Appraisal Tools

**JBI Critical Appraisal: Quasi-Experimental Studies**

1. Is it clear what is the 'cause' and what is the 'effect' (i.e., there is no confusion about which variable comes first)?
2. Were the participants included in any comparisons similar?
3. Were the participants included in any comparisons receiving similar treatment/care, other than the exposure or intervention of interest?
4. Was there a control group?
5. Were there multiple measurements of the outcome both pre and post the intervention/exposure?
6. Was follow-up complete and, if not, were differences between groups in terms of their follow-up adequately described and analysed?
7. Were the outcomes of participants included in any comparisons measured in the same way?
8. Were outcomes measured in a reliable way?
9. Was appropriate statistical analysis used?

| **Study** | **Q1** | **Q2** | **Q3** | **Q4** | **Q5** | **Q6** | **Q7** | **Q8** | **Q9** |
| --- | --- | --- | --- | --- | --- | --- | --- | --- | --- |
| Finan (2012) | Yes | Yes | Yes | No | Yes | Yes | Yes | Yes | Yes |
| Schebesta (2015) | Yes | Yes | Yes | Yes | No | Yes | Yes | Yes | Yes |
| Rumpel (2023) | Yes | Yes | Partially | Yes | No | Yes | Yes | Yes | Yes |
| Samuelson (2016) | Yes | Yes | Yes | Yes | Yes | Yes | Yes | Yes | Yes |
| Jain (2025) | Yes | Partially | N/A | No | Yes | N/A | Yes | Yes | Yes |
| Brettig (2017) | Yes | Yes | Yes | Yes | Yes | Yes | Yes | Yes | Yes |
| Rowe (2002) | Yes | Yes | Yes | Yes | Yes | Yes | Yes | Yes | Yes |

# JBI Critical Appraisal: Analytical Cross-Sectional Studies

1. Were the criteria for inclusion in the sample clearly defined?
2. Were the study subjects and the setting described in detail?
3. Was the exposure measured in a valid and reliable way?
4. Were objective, standard criteria used for measurement of the condition?
5. Were confounding factors identified?
6. Were strategies to deal with confounding factors stated?
7. Were the outcomes measured in a valid and reliable way?
8. Was appropriate statistical analysis used?

| **Study** | **Q1** | **Q2** | **Q3** | **Q4** | **Q5** | **Q6** | **Q7** | **Q8** |
| --- | --- | --- | --- | --- | --- | --- | --- | --- |
| Blackburn (2021) | Yes | Yes | Yes | Yes | Partially | No | Yes | Yes |
| Williams (2010) | Yes | Yes | Yes | Yes | Yes | Yes | Yes | Yes |
| Baker (2016) | Yes | Yes | Yes | Yes | Yes | Yes | Yes | Yes |
| Schalk (2015) | Yes | Yes | Yes | Yes | Yes | No | Yes | Yes |
| Schebesta (2012) | Yes | Yes | Yes | Yes | Yes | No | Yes | Yes |
| Hesselfeldt (2005) | Yes | Yes | Yes | Yes | Yes | Yes | Yes | Yes |
| van Emden (2018) | Yes | Yes | Yes | Yes | Yes | Yes | Yes | Yes |

# JBI Critical Appraisal: Randomized Controlled Trials

1. Was true randomisation used for assignment of participants to treatment groups?
2. Was allocation to treatment groups concealed?
3. Were treatment groups similar at the baseline?
4. Were participants blind to treatment assignment?
5. Were those delivering treatment blind to treatment assignment?
6. Were outcome assessors blind to treatment assignment?
7. Were treatment groups treated identically other than the intervention of interest?
8. Was follow-up complete and if not, were differences between groups in terms of their follow-up adequately described and analysed?
9. Were participants analysed in the groups to which they were randomised?
10. Were outcomes measured in the same way for treatment groups?
11. Were outcomes measured in a reliable way?
12. Was appropriate statistical analysis used?
13. Was the trial design appropriate and any deviations from the standard RCT design (individual randomisation, parallel groups) accounted for in the conduct and analysis of the trial?

| **Study** | **Q1** | **Q2** | **Q3** | **Q4** | **Q5** | **Q6** | **Q7** | **Q8** | **Q9** | **Q10** | **Q11** | **Q12** | **Q13** |
| --- | --- | --- | --- | --- | --- | --- | --- | --- | --- | --- | --- | --- | --- |
| Naik (2001) | Yes | Yes | Yes | Unclear | Yes | Yes | Yes | Yes | Yes | Yes | Yes | Yes | Yes |
| Wong (2019) | Yes | Yes | Yes | Yes | Unclear | Yes | Yes | Yes | Yes | Yes | Yes | Yes | Yes |

# JBI Critical Appraisal: Qualitative Research

- 1. Is there congruity between the stated philosophical perspective and the research methodology?
  2. Is there congruity between the research methodology and the research question or objectives?
  3. Is there congruity between the research methodology and the methods used to collect data?
  4. Is there congruity between the research methodology and the representation and analysis of data?
  5. Is there congruity between the research methodology and the interpretation of results?
  6. Is there a statement locating the researcher culturally or theoretically?
  7. Is the influence of the researcher on the research and vice versa, addressed?
  8. Are participants and their voices adequately represented?
  9. Is the research ethical according to current criteria and is there evidence of ethical approval by an appropriate body?
  10. Do the conclusions drawn in the research report flow from the analysis or interpretation of the data?

| **Author (Year)** | **Q1** | **Q2** | **Q3** | **Q4** | **Q5** | **Q6** | **Q7** | **Q8** | **Q9** | **Q10** |
| --- | --- | --- | --- | --- | --- | --- | --- | --- | --- | --- |
| Kovatch (2020) | Yes | Yes | Yes | Yes | Yes | No | No | Yes | Yes | Yes |

## Appendix 3: Level of Evidence (LoE) and Level of Recommendation (LoR)

## Level of Evidence

| LoE | Criteria |
| --- | --- |
| 1a | Systematic reviews (meta-analysis) containing at least some trials of level 1b evidence, in which results of separate, independently conducted trials are consistent |
| 1b | Randomized controlled trial of good quality and of adequate sample size (power calculation) |
| 2a | Randomized trials of reasonable quality and/or of inadequate sample size |
| 2b | Nonrandomized trials, comparative research (parallel cohort) |
| 2c | Nonrandomized trial, comparative research (historical cohort, literature controls) |
| 3 | Nonrandomized, noncomparative trials, descriptive research |
| 4 | Expert opinions, including the opinion of Work Group members |

## Level of Recommendation (based on LoE analysis of the literature)

| LoR | Criteria |
| --- | --- |
| 1 | Based on one systematic review (1a) or at least two independently conducted research projects classified as 1b |
| 2 | Based on at least two independently conducted research projects classified as level 2a or 2b, within concordance |
| 3 | Based on one independently conducted research project level 2b, or at least two trials of level 3, within concordance |
| 4 | Based on one trial at level 3 or multiple expert opinions, including the opinion of Work Group members (e.g., level 4) |

## LoE and LoR for each study

| Study | LOE |
| --- | --- |
| Finan (2012) | 2c |
| Blackburn (2021) | 2b |
| Naik (2001) | 2b |
| Williams (2010) | 3 |
| Schebesta (2015) | 2c |
| Rumpel (2023) | 2c |
| Kovatch (2020) | 4 |
| Samuelson (2016) | 2b |
| Wong (2019) | 1b |
| Baker (2016) | 2b |
| Schalk (2015) | 2b |
| Schebesta (2012) | 2b |
| Brettig (2017) | 2b |
| Jain (2025) | 3 |
| Rowe (2002) | 2b |
| Hesselfeldt (2005) | 2c |
| van Emden (2018) | 2c |
